# Metabolic Activity in Human Intermuscular Adipose Tissue Directs the Response of Resident PPARγ^+^ Macrophages to Fatty Acids

**DOI:** 10.3390/biomedicines13010010

**Published:** 2024-12-25

**Authors:** Xiaoying Chen, Sebastian Ludger Schubert, Aline Müller, Miguel Pishnamaz, Frank Hildebrand, Mahtab Nourbakhsh

**Affiliations:** 1 Institute of Pathology, RWTH Aachen University Hospital, 52074 Aachen, Germany; xchen@ukaachen.de (X.C.); almueller@ukaachen.de (A.M.); 2 Clinic for Orthopedics, Trauma, and Reconstructive Surgery, RWTH Aachen University Hospital, 52074 Aachen, Germany; seschubert@ukaachen.de (S.L.S.); mpishnamaz@ukaachen.de (M.P.); fhildebrand@ukaachen.de (F.H.)

**Keywords:** PPARγ, macrophages, adipose tissue, fatty acids, mitochondria, VDAC1, COXIV

## Abstract

**Background/Objectives**: Peroxisome proliferator-activated receptor gamma (PPARγ) is a fatty acid-binding transcription activator of the adipokine chemerin. The key role of PPARγ in adipogenesis was established by reports on adipose tissue-resident macrophages that express PPARγ. The present study examined PPARγ^+^ macrophages in human skeletal muscle tissues, their response to fatty acid (FA) species, and their correlations with age, obesity, adipokine expression, and an abundance of other macrophage phenotypes. **Methods**: An ex vivo human skeletal muscle model with surgical specimens that were maintained without or with FAs for up to 11 days was utilized. Immunofluorescence analysis was used to detect macrophage phenotypes and mitochondrial activity. Preconfigured arrays were used to detect the expression of 34 different adipokines and chemokines. **Results**: Data from 14 adults revealed that PPARγ^+^ macrophages exclusively reside in intermuscular adipose tissue (IMAT), and their abundance correlates with the metabolic status of surrounding adipocytes during tissue maintenance in vitro for 9–11 days. Elevated fatty acid levels lead to significant increases in PPARγ^+^ populations, which are correlated with the donor’s body mass index (BMI). **Conclusions**: PPARγ^+^ macrophages represent a distinctly specialized population of regulatory cells that reside within human IMATs in accordance with their metabolic status. Thus, future in-depth studies on IMAT-resident PPARγ^+^ macrophage action mechanisms will elucidate the role of skeletal muscle in the pathogenesis of human metabolic dysfunction.

## 1. Introduction

A critically important role of skeletal muscles is the regulation of metabolism and energy storage. Skeletal muscles are essentially composed of muscle fibers and intermuscular adipose tissue (IMAT), an ectopic lipid and energy deposit that intersperses between myofibers or adjacent bundles of myofibers. IMATs accumulate alongside visceral and subcutaneous adipose tissues in obese individuals. Previous studies have shown that excessive IMAT accumulation is an important pathological hallmark of many human inflammatory myopathies and metabolic diseases, such as insulin resistance and diabetes [1]. Moreover, IMAT RNA-seq analyses have revealed a unique gene expression pattern that is related to insulin sensitivity and absent in other types of adipose tissue, e.g., subcutaneous adipose tissue [2].

The primary function of adipose tissues is the storage and release of metabolic energy by regulating lipogenesis, lipolysis, thermogenesis, and adipocyte mitochondrial function. However, the link between lipid metabolism and mitochondrial functions has not yet been well studied in different human adipose tissues. In mice, simultaneous inhibition of lipolysis and promotion of lipogenesis lead to increased expression of the cytochrome c oxidase IV (COXIV) mitochondrial biomarker [3]. The prevention of diet-induced obesity in rats inhibits the expression of the voltage-dependent anion channel 1 (VDAC1) protein, which is a component of voltage-dependent channels in the outer mitochondrial membrane where hydrophilic molecules diffuse [4]. In addition to lipid metabolism, adipose tissues are also associated with inflammation by hosting functionally specialized resident macrophages, known as adipose tissue macrophages (ATMs), which drive tissue inflammation via the secretion of inflammatory mediators [5,6]. In contrast to circulating monocytes, however, ATMs mostly develop from progenitor cells in the fetal stage and can proliferate and differentiate within the host adipose tissue [7]. Although the total number of ATMs is comparable among individuals, their phenotypes can significantly vary among different age groups, suggesting an adaptive strategy to changing environmental conditions [8]. Accordingly, ATMs that reside in IMAT are assumed to be exclusively involved in the regulatory roles of skeletal muscle in insulin sensitivity and metabolism.

Human blood- or bone marrow-derived macrophages have been previously categorized in proinflammatory M1 and the anti-inflammatory M2 phenotypes, leading to the identification of additional macrophage markers [9]. The most frequently studied human macrophage markers include CD80 and MARCO in M1 or CD163 and CD206 in M2 phenotypes [9]. However, tissue-resident macrophages (TRMs) were shown to produce a variety of overlapping M1 and M2 phenotypes in different tissues, suggesting that M1/M2 classification does not represent the activity of these cells [10]. Multiple genes expressed by monocyte-derived macrophages have been used for profiling of TRMs, but a universal TRM marker is still lacking [11]. Studies on human pancreatic, lung, dermal, adipose, perivascular, and liver tissues have established several TRM markers, including CD11c, CD80, CD163, CD206, MARCO, and PTGER3 [12,13].

Peroxisome proliferator-activated receptor γ (PPARγ) is a ligand-inducible transcription factor that has been implicated in adipogenesis and angiogenesis by increasing the expression of adipokines [14,15,16,17,18,19]. PPARγ has been reported to activate chemerin gene expression by binding to its response elements in the chemerin promoter [16,20,21,22,23]. Moreover, previous binding competition studies have shown that human PPARγ is a decisive receptor for mono- and polyunsaturated fatty acids (FAs), as well as eicosanoids, suggesting its nutritional and clinical implications [17,18]. Furthermore, PPARγ has been reported to enhance the differentiation of murine mesenchymal stem cells into adipocytes in vitro, possibly by regulating FA storage and glucose metabolism [24]. Different PPARγ knockout mouse models have been employed to study PPARγ functions in vivo [15,19]. Constitutive whole-body PPARγ knockouts are deficient in ectopic muscle adipogenesis and myogenic differentiation of skeletal muscle stem cells, referred to as satellite cells [19]. Macrophage-specific knockouts have revealed the importance of PPARγ in macrophage function and protection against the metabolic consequences of obesity by maintaining insulin sensitivity and glucose tolerance [15]. Accordingly, PPARγ expression has been well recognized as a characteristic of ATMs that proliferate locally and perform tissue niche-specific functions in the liver, peritoneum, alveolar, intestinal, and skeletal muscle, or simply in abdominal fat tissue in mouse models [25].

Human IMAT is an understudied and unknown adipose tissue depot, most likely due to its limited accessibility. Therefore, limited information is available considering the abundance of PPARγ-expressing (PPARγ^+^) macrophages in human IMAT and their response to changing metabolic conditions. To address these questions, the present study employed a previously reported human ex vivo model using native skeletal muscle tissue from reconstructive surgeries [26]. The present experimental study reports the distribution of PPARγ^+^ macrophages in human IMATs and their temporal alterations in response to different species of FAs.

## 2. Materials and Methods

### 2.1. Handling and Maintaining Human Tissue Samples

Tissue samples were collected from 14 donors who received surgical treatments at the Clinic for Orthopedics, Trauma, and Reconstructive Surgery of RWTH University Hospital in Aachen, Germany. The excised tissue was transferred into a sterile container and sent to the laboratory immediately. The tissue samples were equally dissected into 18 mm^3^ sections. One section was desaturated in 4% formaldehyde (Otto Fischar GmbH, Saarbrucken, Germany) as a control. The remaining sections were independently placed in a gel mixture consisting of 1% low-melt agarose (Carl Roth GmbH, Karlsruhe, Germany), 10% fetal bovine serum (PAN Biotech GmbH, Aidenbach, Germany), 100 U/mL penicillin, and 100 U/mL streptomycin (PAN Biotech GmbH) in DMEM 2X (Biological Industries, Kibbutz Beit-Haemek, Israel) at 40 °C. DMEM/F-12 medium (Gibco, Thermo Fisher, Waltham, MA, USA), containing 10% fetal bovine serum, 100 U/mL penicillin, and 100 U/mL streptomycin, was added on top of the solidified gel. FAs (FA 1208, 1024, 1014, and 1020, Biotrend Chemikalien GmbH, Cologne, Germany) were dissolved at a 1:2.5 ratio in 10% bovine serum albumin (BSA) in PBS and added to the gel and liquid medium at a final concentration of 50 μM, where indicated. The sections were maintained at 37 °C with 5% CO_2_ for 9 or 11 days. The tissue samples were then carefully removed from the gel medium and incubated in 4% formaldehyde solution (Otto Fischar GmbH) for 24 h.

### 2.2. Quantification of Adipokine and Chemokine Expression in Tissue Samples

The expression of 34 human adipokines and chemokines was analyzed in tissue extracts prepared via preconfigured ProcartaPlex assays (EPXR340-12167-901, Thermo Fisher Scientific, Waltham, MA, USA) according to the manufacturer’s instructions. All extracts were analyzed in at least 4 independent assays, and the mean values were calculated as the final adipokine and chemokine concentration before statistical evaluation.

### 2.3. Hematoxylin and Eosin (HE) Staining

After dehydration in ascending alcohol concentrations (70, 96, and 100%) and xylene, the tissue sections were embedded in paraffin blocks and cut into 5 μm slices via a SLIDE4003E microtome (pfm Medical, Cologne, Germany). The slices were attached to glass slides, deparaffinized, and processed in an automated slide-staining station (Gemini, Thermo Fisher, Waltham, MA, USA) by staining in hematoxylin for 5–10 min, washing with warm water for 10 min, staining with 0.3% eosin for 5 min, and washing again with distilled water. After dehydration in increasing alcohol concentrations (70, 96, and 100%), the slides were treated with xylene and sealed with glass cover slips.

### 2.4. Immunofluorescence (IF) Detection of Human Macrophage Markers

The paraffin slides were heated in citrate buffer (pH 6.0) for 30 min, washed in water, and then washed twice in 0.1% Tween 20 (9127.1, Carl Roth) in PBS. Deparaffinized slides were then blocked in UltraCruz Blocking Reagent (Santa Cruz Biotechnology, Dallas, TX, USA) for 60 min. Slides were incubated with the following diluted primary antibodies in 3% BSA in PBS overnight at 4 °C: anti-human PPARγ (1:500, MA5-14889, Thermo Fisher Scientific), CD80 (1:1000, ab134120, Abcam, Cambridge, UK), CD11c (1:100, ab52632, Abcam), MARCO (1:100, PA5-64134, Thermo Fisher Scientific), CD163 (1:200, ab156769, Abcam), CD206 (1:100, PA5-101657, Thermo Fisher Scientific), PTGER3 (1:100, PA5-102057, Thermo Fisher Scientific), or VDAC1 (1:100, ab154856, Abcam). The slides were washed and stained with anti-rabbit IgG488 (ab150081, Abcam), anti-rabbit IgG594 (ab150084, Abcam), or anti-mouse IgG488 (ab150117, Abcam) diluted 1:200 in UltraCruz Blocking Reagent for 60 min. After washing, the slides were incubated with 0.1% DAPI (D9542, Sigma–Aldrich, St. Louis, MO, USA) in PBS for 5 min and then sealed with glass coverslips in Immu-Mount (9990402, Thermo Fisher Scientific).

### 2.5. HE and IF Imaging

HE and IF images were obtained via an automated microscope with an integrated camera (DM6000B, Leica Microsystems, Wetzlar, Germany). Of note, 340–380 nm, 450–490 nm, and 590 nm filters were used for the DAPI, anti-rabbit or anti-mouse IgG488, and anti-rabbit IgG594 images, respectively. The images were processed and merged via Diskus software version 10 (Leica). At least 2 random fields of view (0.24 mm^2^) in cross-sectional SMFs or IMATs were selected in independently stained sections to determine the number of positive cells. SMFs and IMATs were evaluated selectively. All other areas of known or unknown function were strictly excluded throughout the evaluation process. Average VDAC1 expression was estimated via integrated signal intensities normalized to the size of selected areas and the number of implicated muscle fibers or adipocytes as indicated via ImageJ software v1.54g (https://imagej.net/, accessed on 11 October 2024).

### 2.6. Statistical Data Analysis

All the statistical tests in the present study were performed via GraphPad Prism (version 10.1.2, GraphPad Software, San Diego, CA, USA) and are described individually in the figure legends. Briefly, the Shapiro–Wilk test was applied to examine the normality of the distribution of the variables. Pearson’s or Spearman’s rank correlation analyses were performed between the variables, e.g., cell number, adipokine expression, chemokine expression, and clinical characteristics. The significance levels of the relative fold changes were calculated via one-sample *t*-tests or the Wilcoxon signed-rank test. The significance of differences in cell counts before and after tissue maintenance was analyzed via a paired *t*-test or the Wilcoxon signed-rank test. In all the experiments, *p*-values ≤ 0.05 were considered statistically significant.

## 3. Results

Human intermuscular adipose tissue (IMAT) and its cellular components are still understudied areas of research, most likely due to challenging ethical and logistical requirements. In the present study, skeletal muscle tissue specimens were collected from 14 adults who received surgical treatments and provided signed informed consent. The present study included all donors who were medically fit and provided adequate quality and quantity of skeletal muscle tissue samples. No other exclusion criteria were applied. The study group age ranged from 22 to 82 years, including eleven females and three males, as well as two individuals diagnosed with type 2 diabetes (T2D) (Supplementary Materials, Table S1). The body mass index (BMI) of the study participants ranged between 20.3 and 46.9 kg/m^2^ (Supplementary Materials, Table S1).

### 3.1. Steady-State Density of PPARγ^+^ Macrophage Populations in Human IMATs

**Figure 1:**
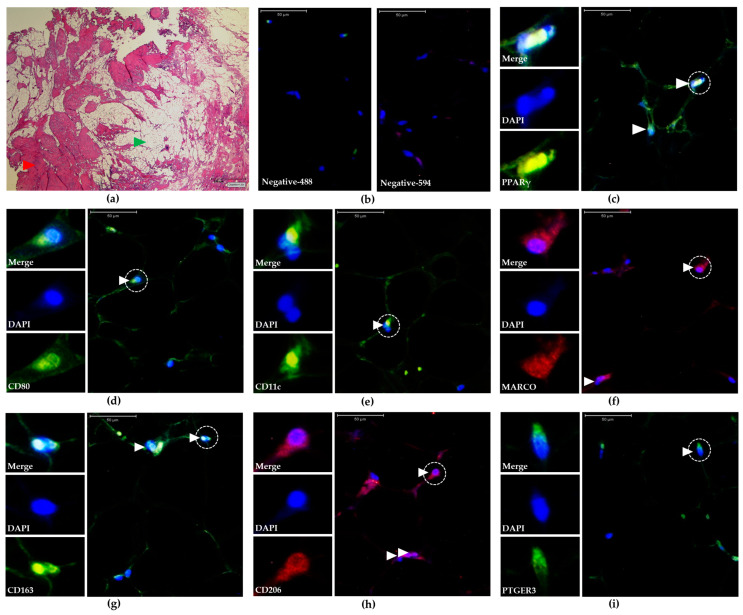
Representative images of skeletal muscle tissue (HE) and IMATs (IF). All images were obtained from Participant P6. (**a**) HE image showing the human skeletal muscle tissue comprising the areas of SMFs (red arrowhead) and IMATs (green arrowhead). The scale bar (lower right) indicates 1000 µm. (**b**) IF images were obtained after costaining with DAPI and secondary antibodies as negative controls (negative-488 or 594). The scale bars (upper left) indicate 50 µm. (**c**–**i**) IF images of IMATs after costaining with primary antibodies against designated human markers (white, lower left) and the corresponding secondary antibodies and DAPI. The small panels on the left side represent magnified single-cell images labeled with dashed line circles in larger images using IgG488 (green), IgG594 (red), and DAPI (blue) filters. DAPI and IgG594 or DAPI and IgG488 were merged (Merge) to determine the specificity of the detected signals. The white arrowheads indicate verified positive macrophages. The scale bars (upper left) indicate 50 µm.

**Figure 2:**
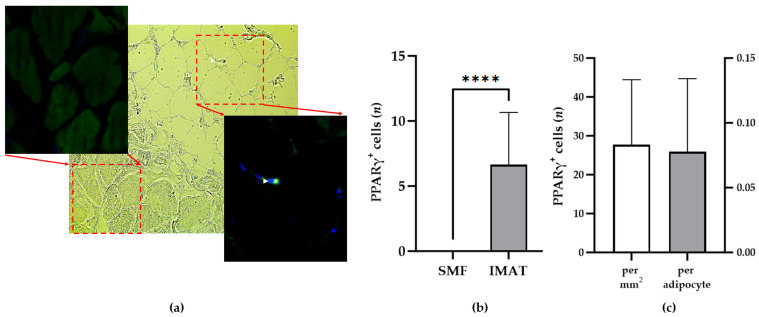
PPARγ^+^ macrophages exclusively reside in IMATs. (**a**) Representative images from a skeletal muscle tissue slice from P2 after IF staining using a primary antibody against PPARγ, IgG488-labeled secondary antibody, and DAPI. The image of brightfield microscopy (middle panel) comprises skeletal muscle fibers (left) and intermuscular adipose tissue with adipocytes (right). Magnified IF images show the labeled areas of skeletal muscle fibers (upper left panel) and intermuscular adipose tissue (lower right panel) exposing a PPARγ^+^ macrophage (white arrowhead), respectively. (**b**) The diagram shows the mean number of PPARγ^+^ macrophages (*y*-axis) in the IMAT and SMF fields of 0.24 mm^2^ (*x*-axis) in donor tissue samples (n = 14). (**c**) The diagram shows the mean number of PPARγ^+^ macrophages (*y*-axis) relative to 1 mm^2^ of IMATs (left *y*-axis) or relative to the number of adipocytes in 1 mm^2^ of IMATs in donor tissue samples (n = 14). The Mann–Whitney test was used to assess the significance of differences in the number of PPARγ^+^ macrophages between SMFs and IMATs. *p* ≤ 0.0001 (****).

To obtain broad data on the population of PPARγ^+^ macrophages that reside in the native environment of human skeletal muscle tissue, skeletal muscle tissue samples were collected and dissected in a series for detailed analysis immediately after surgery and for later experimental studies. One sample from each participant was preserved in paraffin and sectioned on slides for further analysis of resident macrophages. One tissue slide was subjected to HE staining to determine the location, size, and scattering of IMATs and SMFs (Figure 1a). The remaining tissue slides were subjected to IF staining with primary antibodies against human CD80, CD11c, CD163, CD206, MARCO, PTGER3, or PPARγ, followed by staining with fluorescently labeled secondary antibodies and DAPI (Figure 1c–i). Control experiments using secondary antibodies alone were performed to isolate background signals (Figure 1b). The specificity of positively detected cells was further verified by comparison with DAPI signals. Overall, 98 IF images were analyzed from 14 donors to determine the number of IMATs and SMFs with different macrophage phenotypes immediately after surgery. Two randomly selected microscopic fields of IMAT or SMF views (0.24 mm^2^) were evaluated, rendering mean numbers of CD80^+^, CD11c^+^, CD163^+^, CD206^+^, MARCO^+^, PTGER3^+^, and PPARγ^+^ macrophages within the IMATs or SMFs from individual participants. PPARγ^+^ macrophages were exclusively detected in IMATs from all participants (Figure 2a,b). Moreover, the density of PPARγ^+^ macrophages in IMATs related to either the tissue area or the number of adipocytes was not significantly different among the participants (Figure 2c).

As reported previously, CD80^+^, CD11c^+^, CD163^+^, CD206^+^, MARCO^+^, and PTGER3^+^ macrophages were detected within the IMATs and SMFs of all participants [26]. Next, the present study investigated possible correlations between the abundance of PPARγ^+^ macrophages and other macrophage phenotypes, donor age, or BMI (Table 1). Table 1 shows that there were no significant correlations (r) with *p*-values ≥ 0.0973, suggesting that PPARγ^+^ macrophages may constitute an independent population of IMAT-resident macrophages. Similarly, the abundance of PPARγ^+^ macrophages did not correlate with other SMF-resident macrophages, despite a negligible correlation with CD80^+^ and MARCO^+^ macrophages (Supplementary Materials, Table S2).

### 3.2. The Number of PPARγ^+^ Macrophages Is Independent of Mitochondrial Activity in IMAT

**Figure 3:**
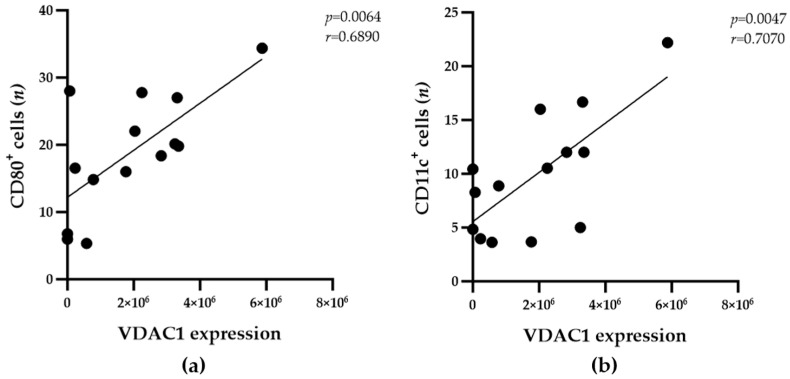
The numbers of CD80^+^ and CD11c^+^ macrophages correlate with adipocyte VDAC1 expression in the IMATs of donor samples. Pearson correlation analyses were employed to determine the relationships between the mean VDAC1 expression levels and the mean numbers of CD80^+^ (**a**) and CD11c^+^ (**b**) macrophages in 0.24 mm^2^ of IMATs from the donors (n = 14). The correlation coefficients (r) and significance levels (*p*) for the relationships are presented at the top right of each diagram.

The VDAC1 and COXIV mitochondrial proteins are markers of the metabolic activity of cells both in vitro and in vivo. In the present study, tissue slides from all participants were subjected to IF staining using primary antibodies against human VDAC1 or COXIV and DAPI. The mean expression of VDAC1 and COXIV protein per adipocyte was estimated by calculating the integrated fluorescence signal intensity within the 28 corresponding IF images. The statistical analysis revealed no correlations between the number of IMAT-resident PPARγ^+^ macrophages and the expression levels of VDAC1 or COXIV in IMAT adipocytes (Table 2). In contrast, the CD80^+^ and CD11c^+^ macrophage populations were significantly correlated with VDAC1 expression levels, suggesting a possible link to metabolic activity in IMATs (Figure 3a,b).

### 3.3. The Number of PPARγ^+^ Macrophages Is Independent of Adipokines/Chemokines Expression

**Figure 4:**
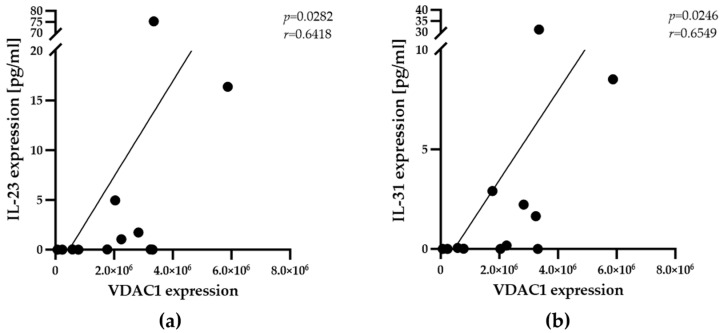
The expression levels of IL-23 and IL-31 correlate with adipocyte VDAC1 expression in the IMATs of donor samples. Spearman’s rank correlation analyses were employed to determine the relationships between mean VDAC1 and IL-23 (**a**) or IL-31 (**b**) expression levels (n = 12). The correlation coefficients (r) and significance levels (*p*) for the relationships are presented at the top right of each diagram.

Next, the present study analyzed whether the population of PPARγ^+^ macrophages is correlated with the overall expression of adipokines or inflammatory factors. A predesigned antibody array was utilized for the detection of 9 adipokines, including IL-1 beta, IL-10, IL-18, IL-4, IL-6, IL-8, MCP-1 (CCL2), stromal cell-derived factor (SDF)-1 alpha, and TNF alpha adipokines, as well as 25 chemokines, including eotaxin (CCL11), granulocyte-macrophage colony-stimulating factor (GM-CSF), growth-regulated protein (GRO) alpha (CXCL1), interferon (IFN) alpha, IFN gamma, IL-1 alpha, IL-12p70, IL-13, IL-15, IL-17A (CTLA-8), IL-1RA, IL-2, IL-21, IL-22, IL-23, IL-27, IL-31, IL-5, IL-7, IL-9, IFN gamma-induced protein (IP)-10 (CXCL10), macrophage inflammatory protein (MIP)-1 alpha (CCL3), MIP-1 beta (CCL4), regulated on activation, normal T-cell expressed and secreted (RANTES) (CCL5), and TNF beta. A minimum amount of 100 mg of native muscle tissue was required to prepare protein extracts for analysis. Two participants (P2 and P12) provided a limited sample size, which was not enough for protein extraction. The extracts from the other 12 participants were subjected to four independent experiments, and the results were used to obtain the mean values of the expression levels. Overall, the expression levels of adipokines in skeletal muscle samples were not significantly different and were generally lower than those of chemokines. Further analysis of the data revealed no correlations between the expression levels of chemokines (Supplementary Materials, Table S3) or adipokines and the number of PPARγ^+^ macrophages (Table 3). However, there were significant correlations between the overall expression of IL-23 and IL-31 and the metabolic activity of IMAT adipocytes, as determined by VDAC1 expression (Figure 4a,b and Supplementary Materials, Table S4).

### 3.4. The Metabolic Activity of Adipocytes in the IMAT and the Number of PPARγ^+^ Macrophages Increase During Tissue Maintenance In Vitro

**Figure 5:**
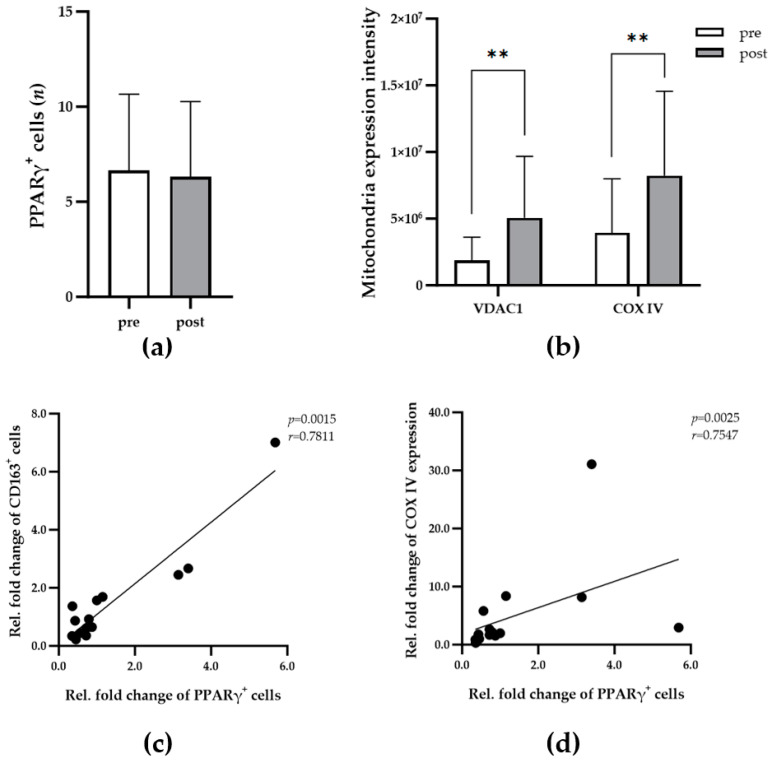
Dynamics of the PPARγ^+^ macrophage population in IMATs during maintenance in vitro. (**a**) The diagram shows the mean number of PPARγ^+^ macrophages (*y*-axis) in 0.24 mm^2^ of IMAT from all participants (n = 14) before (pre, white bar) and after (post, gray bars) tissue maintenance in vitro. (**b**) The diagram shows the mean expression of VDAC1 and COXIV (*y*-axis) in 0.24 mm^2^ of IMAT from all participants (n = 14) before (pre, white bars) and after (post, gray bars) tissue maintenance in vitro. A paired t-test or Wilcoxon signed-rank test was applied to evaluate the significance of differences before and after cultivation. *p* ≤ 0.01 (**). (**c**,**d**) Spearman’s rank correlation analyses were applied to determine the relationships between the mean number of PPARγ^+^ macrophages and the mean number of CD163^+^ (**c**) or the expression level of COXIV (**d**) in 0.24 mm^2^ of IMAT from all donors (n = 14). The correlation coefficients (r) and significance levels (*p*) for the relationships are presented at the top right of each diagram.

For the temporal study of PPARγ^+^ macrophage populations, the initial surgical samples as described above (Section 2.1) were maintained in vitro for 9 or 11 days, and the number of PPARγ^+^ macrophages was then determined. The comparison of PPARγ^+^ macrophages in all samples before (pre) and after maintenance in vitro (post) revealed no significant changes in the number of positive cells (Figure 5a). Further analysis of the mean VDAC1 and COXIV expression levels in the IMATs of all participants (56 IF images) revealed a significant increase, indicating increased mitochondrial activity in adipocytes under maintenance conditions (Figure 5b). For direct comparison, the relative fold change in variables (positive cells, adipokines, chemokines, VDAC1, or COXIV expression) was calculated by normalizing the post-maintenance values to the corresponding premaintenance values. Thus, a relative fold change greater than 1 indicates an increase in cell number or protein expression after maintenance in vitro (Figure 5c,d). The results revealed significant correlations between the relative fold changes in PPARγ^+^ and CD163^+^ macrophages or COXIV expression (Figure 5c,d).

### 3.5. U-FA and S-FA Increase the Number of PPARγ^+^ Macrophages Without Affecting Mitochondrial Activity in IMATs

**Figure 6:**
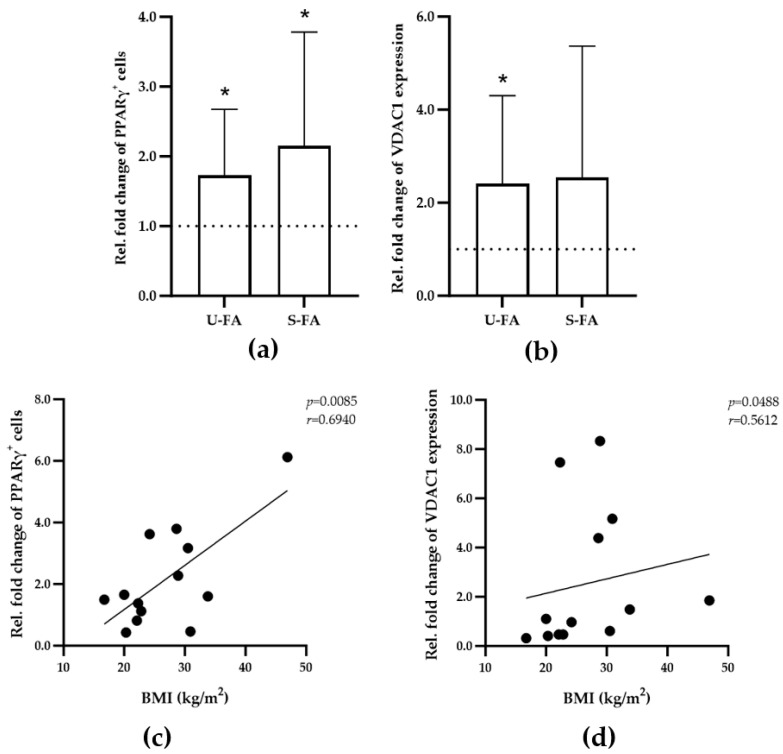
Dynamics of the PPARγ^+^ macrophage population in IMATs in response to S-FAs and U-FAs during maintenance in vitro. (**a**) The diagram shows the relative fold change in PPARγ^+^ macrophage numbers (*y*-axis) in 0.24 mm^2^ of IMAT from all participants (n = 14) in response to U-FA or S-FAs before (*x*-axis) in vitro culture. (**b**) The diagram shows the relative fold change in the expression of VDAC1 (*y*-axis) in 0.24 mm^2^ of IMAT from all participants (n = 14) in response to U-FA or S-FAs before (*x*-axis) in vitro culture. One-sample *t*-tests or Wilcoxon signed-rank tests were used to assess the significance of differences before and after cultivation. *p* ≤ 0.05 (*). (**c**,**d**) Pearson correlation and Spearman’s rank correlation analyses were employed to determine the relationships between the S-FA-mediated relative fold change in the number of PPARγ^+^ macrophages ((**c**), *y*-axis) or the relative fold change in the expression of VDAC1 ((**d**), *y*-axis) and donor BMI (n = 14). The correlation coefficients (r) and significance levels (*p*) for the relationships are presented at the top right of each diagram.

Saturated and unsaturated FAs (S-FAs and U-FAs, respectively) with 16 or 18 carbon atoms (C16 or C18) are high-affinity ligands of PPARγ [17,18] and lead to the accumulation of distinct TRM phenotypes in human skeletal muscle tissue [26]. Thus, the present study examined the effects of saturated C16, saturated C18, monounsaturated C16 (C16[1]), and di-unsaturated C18 (C18[2]) on the number of PPARγ^+^ macrophages and the expression of VDAC1 or COXIV in IMATs. A series of dissected skeletal muscle tissues from all participants were maintained without or with 50 µM concentration of S-FAs (C16 or C18) or U-SFAs (C16[1] or C18[2]) for 9 or 11 days in vitro. The PPARγ^+^ macrophage number, VDAC1 expression, and COXIV expression were then analyzed as described above in 168 IF images from 14 donors. All FAs led to a significant accumulation of PPARγ^+^ macrophages (Figure 6a) and VDAC1 expression (Figure 6b), which was used as a measure of mitochondrial activity. Notably, COXIV expression was not significantly affected by any FA (Supplementary Materials, Figure S1). Furthermore, examination of the participants’ characteristics revealed that the responses to S-FAs were significantly correlated with the participants’ BMI (Figure 6c,d). Together, these results suggested that the response of IMAT-resident PPARγ^+^ macrophages to S-FAs increases with BMI and obesity. However, this suggestion needs to be confirmed in future studies involving a higher number of participants.

## 4. Discussion

Despite compelling evidence for a strong link between metabolic diseases and IMAT expansion, the cellular components of human IMATs and their role in metabolic pathways remain unexplored. The presented human skeletal muscle tissue model provides a straightforward strategy for the study of the cellular components of IMATs and their response to different conditions. The present study demonstrated the exclusive association of PPARγ^+^ macrophages with metabolically active adipocytes in human IMATs and their immediate accumulation in response to elevated levels of fatty acids within the native tissue environment. Future inclusion of diseased donor groups in the present study model has the potential to fully reveal the impact of IMAT cellular components and secreted factors on human health and diseases.

The study of animal knockout models has provided important insights into the possible functions of PPARγ in macrophages in adipose tissue [27,28,29]. Recent studies on the activation of TRM signaling pathways suggested a lipid-sensing role for PPARγ [30,31]. Only a few human studies have investigated PPARγ^+^ macrophages in visceral or subcutaneous adipose tissue and hypothesized novel regulatory roles for PPARγ in human diseases [32,33]. The present study revealed that PPARγ^+^ macrophages are a constitutive and exclusive component of IMATs in all studied donors (Figure 2a,b), including those diagnosed with T2D or whose BMIs were higher than the normal range (≥25 kg/m^2^) (Supplementary Materials, Table S1). The PPARγ^+^ macrophage populations did not correlate with other macrophage phenotypes in IMATs or SMFs, except for a negligible negative correlation with SMF-resident CD80^+^ and MARCO^+^ macrophages (Supplementary Materials, Table S2). Moreover, there were no significant differences in the number of resident PPARγ^+^ macrophages among the donors. These observations imply that PPARγ^+^ macrophages represent a distinct minor population in human IMATs, possibly with a unique regulatory function. However, some limitations of the present study should be considered. First, the sex and age variables of the participants in the study group were not normally distributed (Supplementary Materials, Table S1). Thus, the characteristics of IMAT-resident PPARγ^+^ macrophage populations should be further verified in future studies by recruiting young male participants. Second, like all histological studies, the present study relied on a limited set of established markers and antibodies for detecting human macrophages. Accordingly, many other relevant but still unestablished phenotypes remain to be characterized and may play important roles in human skeletal muscle tissue. Third, IF staining is efficient in capturing the spatiotemporal dynamics of PPARγ^+^ macrophage populations in IMATs but may not detect small variations in PPARγ expression levels. Thus, the FA-mediated increase in PPARγ^+^ macrophages most likely corresponds to a distinct PPARγ expression level that was significantly lower and undetectable in a subpopulation of macrophages before FA stimulation. Importantly, PPARγ^+^ macrophages were randomly scattered within the IMATs after FA stimulation. This pattern opposes local cell expansion. Nevertheless, PPARγ expression is a relevant hallmark of an exclusive population of IMAT-resident macrophages that significantly respond to fatty acids.

A critical role of PPARγ in metabolic regulation has been implied from previous clinical studies showing the protective effects of human PPARγ gene polymorphisms on obesity and T2D [32]. The present study did not identify any clues for parallels between the number of PPARγ^+^ macrophages in the IMATs of participants and adipokine expression or adipocyte metabolic activity via VDAC1 and COXIV expression (Figure 2, Table 2 and Table 3). This may be partially due to the low expression levels of adipokines in human skeletal muscle tissue. Moreover, the detected level of cytokines displays overall expression in the complete tissue and not exclusively in the IMAT, which is a limitation of expression analysis. Nevertheless, the marked correlations between VDAC1 expression and IL-23, IL-31, CD80^+^, or CD11c^+^ macrophages confirmed the ability of the present model to uncover relevant metabolic correlations. Accordingly, IL-23 and IL-31 have been implicated in cellular metabolism [33,34], and CD80^+^ and CD11c^+^ macrophages are negatively correlated with mitochondrial and metabolic dysfunction, respectively [35,36]. Interestingly, excess culture medium during tissue maintenance significantly increased mitochondrial activity in adipocytes, which was correlated with the number of PPARγ^+^ macrophages. Despite many efforts, the level of adipokine and chemokine expression after maintenance in vitro remains unknown because of the small sample size. Although the mean number of PPARγ^+^ macrophages of all participants did not change after maintenance in vitro, there was a significant increase in PPARγ+ macrophage number in three participants, namely Participants P1, P10, and P11, with a healthy range of BMIs between 20 and 22.3 kg/m^2^ (Supplementary Materials, Figure S2). Moreover, the number of PPARγ^+^ macrophages correlated with increased COXIV expression after maintenance in vitro. These observations led to the conclusion that the abundance of PPARγ^+^ macrophages depends on the metabolic status of skeletal muscle tissue.

The most relevant feature of the PPARγ^+^ macrophage populations was their common response to FAs. Compared with the scattered effects mediated by culture medium, U-FAs and S-FAs led to a significant increase in PPARγ^+^ macrophages in IMATs of all donors. By binding FAs, the PPARγ protein was reported to act as a transcription factor and instantly activate the expression of downstream adipokines, such as chemerin [14,15,16,17,18,19,20,21,22,23]. However, the increase in the number of PPARγ^+^ macrophages in IMATs described here is rather a long-term process that most likely involves increased expression of PPARγ downstream genes. This assumption was further confirmed by the significant positive correlation between the S-FA-mediated increase in PPARγ^+^ macrophages and donor BMI values. These observations emphasize a positive feedback loop of increased adipocytes, FAs, PPARγ, PPARγ^+^ macrophages, and their downstream gene products, likely adipokines.

## 5. Conclusions

The human skeletal muscle tissue model facilitated the characterization and temporal study of resident macrophage populations under experimental conditions. Using this study model, we demonstrated that PPARγ^+^ macrophage populations exclusively reside in the IMATs from all studied donors and represent a distinct phenotype of regulatory cells. Their abundance and FA response strongly differed from other macrophage phenotypes in all donors. Most importantly, in vitro stimulation with FAs increased the number of PPARγ^+^ macrophages in IMATs. This effect was more pronounced by S-FAs. These exceptional features of PPARγ^+^ macrophages likely establish the mechanism underlying the pathogenesis of human metabolic dysfunction in obesity, which is associated with excessive IMAT accumulation. Thus, future in-depth studies on the mechanisms of PPARγ^+^ macrophage action in human skeletal muscle specimens will provide guidance for the development of new strategies for the treatment of human metabolic diseases.

## Figures and Tables

**Table 1:** Correlations between the number of PPARγ^+^ macrophages and donor characteristics or other macrophages, as calculated via Spearman’s rank correlation analyses.

		Characteristics		Macrophages					
		Age	BMI	CD80^+^	CD11c^+^	MARCO^+^	CD163^+^	CD206^+^	PTGER3^+^
PPARγ^+^	*p*	0.1180	0.8201	0.9638	0.1634	0.5629	0.0973	0.9152	0.9758
	r	−0.4383	0.0681	0.01544	0.3938	0.1692	0.4637	0.03311	−0.01099

The significance levels (*p*) and correlation coefficients (r) are presented.

**Table 2:** Correlations between the number of PPARγ^+^ macrophages and adipocyte VDAC1 or COXIV expression levels, as calculated via Spearman’s rank correlation analyses.

		VDAC1	COX IV
PPARγ^+^	*p*	0.8557	0.6158
	r	0.05495	−0.1473

The significance levels (*p*) and correlation coefficients (r) are presented.

**Table 3:** Correlations between the number of PPARγ^+^ macrophages and the expression of adipokines in 12 donors, as calculated via Spearman’s rank correlation analyses.

		*p*	r
PPARγ^+^	IL-1 beta	0.6785	−0.1331
	IL-18	0.2464	−0.3636
	IL-4	0.8004	0.0839
	IL-6	0.7160	−0.1189
	IL-8 (CXCL8)	0.2869	−0.3357
	MCP-1 (CCL2)	0.3424	−0.3007
	SDF-1 alpha	0.1767	−0.4196
	TNF alpha	0.2831	0.3380

The significance levels (*p*) and correlation coefficients (r) are presented.

## Data Availability

The original contributions presented in this study are included in the article/Supplementary Materials. Further inquiries can be directed to the corresponding author.

## Supplementary Materials

The supporting information can be downloaded at https://www.mdpi.com/article/10.3390/biomedicines13010010/s1. Table S1. Participant characteristics; Table S2. Correlations between the numbers of PPARγ+ macrophages in IMATs and other macrophage phenotypes in SMFs (upper row) of 14 donors calculated using Spearman’s rank analyses; Table S3. Correlations between the number of PPARγ+ macrophages and the expression of 23 chemokines in skeletal muscle tissue samples from12 donors were calculated using Spearman’s rank analyses; Table S4. Correlations between the mean expression level of VDAC1 in IMATs from 12 donors and the expression of 31 adipokines (upper panel)/chemokines (lower panel) in skeletal muscle tissue samples from 12 donors were calculated using Spearman’s rank analyses. Figure S1. The diagram shows the relative fold change in expression of COXIV (*y*-axis) in 0.24 mm^2^ IMATs of all participants (n = 14) in response to U-FA or S-FAs (*x*-axis) in vitro. A series of dissected skeletal muscle tissues from all participants were maintained with or without 50 µM concentration of S-FA (C16 or C18) or U-SFA (C16[1] or C18[2]) for 9 or 11 days in vitro. The COXIV expression was analyzed after IF staining with primary antibodies against COXIV followed by staining with fluorescently labeled secondary antibodies and DAPI. The relative fold change in COXIV expression was calculated by normalizing the post-maintenance values to the corresponding premaintenance values. One sample t test and Wilcoxon signed-rank test were employed to assess the level of significance *p* ≥ 0.29; Figure S2. The diagram shows the mean number of PPARγ+ macrophages (*y*-axis) in 0.24 mm^2^ IMATs of participants 1, 10, and 11 (n = 3) before (pre, white bar) and after (post, gray bars) tissue maintenance in vitro. One sample from each participant was preserved in paraffin and sectioned on slides (pre) and additional samples from all donors were maintained in vitro (post). The number of PPARγ+ macrophages were determined after IF staining with primary antibodies against human PPARγ, followed by staining with fluorescently labeled secondary antibodies and DAPI. Two randomly selected microscopic fields of IMAT views (0.24 mm^2^) were evaluated, rendering mean numbers of PPARγ+ macrophages within the IMATs or SMFs from individual participants. The level of significance (p) was analyzed using paired *t* test.

## References

[1] Kewalramani G., Bilan P.J., Klip A. (2010). Muscle insulin resistance: Assault by lipids, cytokines and local macrophages. Curr. Opin. Clin. Nutr. Metab. Care.

[2] Sachs S., Zarini S., Kahn D.E., Harrison K.A., Perreault L., Phang T., Newsom S.A., Strauss A., Kerege A., Schoen J.A. (2019). Intermuscular adipose tissue directly modulates skeletal muscle insulin sensitivity in humans. Am. J. Physiol. Endocrinol. Metab..

[3] Qu Y., Chen S., Zhou L., Chen M., Li L., Ni Y., Sun J. (2022). The different effects of intramuscularly-injected lactate on white and brown adipose tissue in vivo. Mol. Biol. Rep..

[4] Parray H.A., Yun J.W. (2015). Proteomic Identification of Target Proteins of Thiodigalactoside in White Adipose Tissue from Diet-Induced Obese Rats. Int. J. Mol. Sci..

[5] Nishimura S., Manabe I., Nagasaki M., Hosoya Y., Yamashita H., Fujita H., Ohsugi M., Tobe K., Kadowaki T., Nagai R. (2007). Adipogenesis in obesity requires close interplay between differentiating adipocytes, stromal cells, and blood vessels. Diabetes.

[6] Bourlier V., Bouloumie A. (2009). Role of macrophage tissue infiltration in obesity and insulin resistance. Diabetes Metab..

[7] Mass E., Nimmerjahn F., Kierdorf K., Schlitzer A. (2023). Tissue-specific macrophages: How they develop and choreograph tissue biology. Nat. Rev. Immunol..

[8] Cui C.Y., Driscoll R.K., Piao Y., Chia C.W., Gorospe M., Ferrucci L. (2019). Skewed macrophage polarization in aging skeletal muscle. Aging Cell.

[9] Murray P.J., Allen J.E., Biswas S.K., Fisher E.A., Gilroy D.W., Goerdt S., Gordon S., Hamilton J.A., Ivashkiv L.B., Lawrence T. (2014). Macrophage activation and polarization: Nomenclature and experimental guidelines. Immunity.

[10] Martinez F.O., Gordon S. (2014). The M1 and M2 paradigm of macrophage activation: Time for reassessment. F1000Prime Rep..

[11] Bao Y., Wang G., Li H. (2024). Approaches for studying human macrophages. Trends Immunol..

[12] Dick S.A., Wong A., Hamidzada H., Nejat S., Nechanitzky R., Vohra S., Mueller B., Zaman R., Kantores C., Aronoff L. (2022). Three tissue resident macrophage subsets coexist across organs with conserved origins and life cycles. Sci. Immunol..

[13] Nobs S.P., Kopf M. (2021). Tissue-resident macrophages: Guardians of organ homeostasis. Trends Immunol..

[14] Wolford J.K., Yeatts K.A., Dhanjal S.K., Black M.H., Xiang A.H., Buchanan T.A., Watanabe R.M. (2005). Sequence variation in PPARG may underlie differential response to troglitazone. Diabetes.

[15] Odegaard J.I., Ricardo-Gonzalez R.R., Goforth M.H., Morel C.R., Subramanian V., Mukundan L., Red Eagle A., Vats D., Brombacher F., Ferrante A.W. (2007). Macrophage-specific PPARgamma controls alternative activation and improves insulin resistance. Nature.

[16] Muruganandan S., Parlee S.D., Rourke J.L., Ernst M.C., Goralski K.B., Sinal C.J. (2011). Chemerin, a novel peroxisome proliferator-activated receptor gamma (PPARgamma) target gene that promotes mesenchymal stem cell adipogenesis. J. Biol. Chem..

[17] Kliewer S.A., Sundseth S.S., Jones S.A., Brown P.J., Wisely G.B., Koble C.S., Devchand P., Wahli W., Willson T.M., Lenhard J.M. (1997). Fatty acids and eicosanoids regulate gene expression through direct interactions with peroxisome proliferator-activated receptors alpha and gamma. Proc. Natl. Acad. Sci. USA.

[18] Grygiel-Gorniak B. (2014). Peroxisome proliferator-activated receptors and their ligands: Nutritional and clinical implications--a review. Nutr. J..

[19] Dammone G., Karaz S., Lukjanenko L., Winkler C., Sizzano F., Jacot G., Migliavacca E., Palini A., Desvergne B., Gilardi F. (2018). PPARgamma Controls Ectopic Adipogenesis and Cross-Talks with Myogenesis During Skeletal Muscle Regeneration. Int. J. Mol. Sci..

[20] Silva A.F., Abruzzese G.A., Ferrer M.J., Heber M.F., Ferreira S.R., Cerrone G.E., Motta A.B. (2022). Fetal programming by androgen excess impairs liver lipid content and PPARg expression in adult rats. J. Dev. Orig. Health Dis..

[21] Li Y., Ma W.G., Li X.C. (2021). Identification of Blood miR-216a, miR-377 and Their Target Genes ANGPTL4, GAP-43 and Serum of PPARG as Biomarkers for Diabetic Peripheral Neuropathy of Type 2 Diabetes. Clin. Lab..

[22] Kim J.W., Kim J.H., Lee Y.J. (2024). The Role of Adipokines in Tumor Progression and Its Association with Obesity. Biomedicines.

[23] Abruzzese G.A., Heber M.F., Campo Verde Arbocco F., Ferreira S.R., Motta A.B. (2019). Fetal programming by androgen excess in rats affects ovarian fuel sensors and steroidogenesis. J. Dev. Orig. Health Dis..

[24] Lehmann J.M., Moore L.B., Smith-Oliver T.A., Wilkison W.O., Willson T.M., Kliewer S.A. (1995). An antidiabetic thiazolidinedione is a high affinity ligand for peroxisome proliferator-activated receptor gamma (PPAR gamma). J. Biol. Chem..

[25] Toobian D., Ghosh P., Katkar G.D. (2021). Parsing the Role of PPARs in Macrophage Processes. Front. Immunol..

[26] Chen X., Muller A., Pishnamaz M., Hildebrand F., Bollheimer L.C., Nourbakhsh M. (2024). Differential Fatty Acid Response of Resident Macrophages in Human Skeletal Muscle Fiber and Intermuscular Adipose Tissue. Int. J. Mol. Sci..

[27] Strand E., Lysne V., Grinna M.L., Bohov P., Svardal A., Nygard O., Berge R.K., Bjorndal B. (2019). Short-Term Activation of Peroxisome Proliferator-Activated Receptors alpha and gamma Induces Tissue-Specific Effects on Lipid Metabolism and Fatty Acid Composition in Male Wistar Rats. PPAR Res..

[28] Cho Y., Hazen B.C., Russell A.P., Kralli A. (2013). Peroxisome proliferator-activated receptor gamma coactivator 1 (PGC-1)- and estrogen-related receptor (ERR)-induced regulator in muscle 1 (Perm1) is a tissue-specific regulator of oxidative capacity in skeletal muscle cells. J. Biol. Chem..

[29] Zidek V., Mlejnek P., Simakova M., Silhavy J., Landa V., Kazdova L., Pravenec M., Kurtz T.W. (2013). Tissue-specific peroxisome proliferator activated receptor gamma expression and metabolic effects of telmisartan. Am. J. Hypertens..

[30] Zuo S., Wang Y., Bao H., Zhang Z., Yang N., Jia M., Zhang Q., Jian A., Ji R., Zhang L. (2024). Lipid synthesis, triggered by PPARgamma T166 dephosphorylation, sustains reparative function of macrophages during tissue repair. Nat. Commun..

[31] Weng X., Jiang H., Walker D.J., Zhou H., Lin D., Wang J., Kang L. (2024). Deletion of CD44 promotes adipogenesis by regulating PPARgamma and cell cycle-related pathways. J. Endocrinol..

[32] Butt H., Shabana, Hasnain S. (2016). The C1431T polymorphism of peroxisome proliferator activated receptor gamma (PPARgamma) is associated with low risk of diabetes in a Pakistani cohort. Diabetol. Metab. Syndr..

[33] Wang L., Liu L., Qian W., Zheng Z. (2022). CD5L Secreted by Macrophage on Atherosclerosis Progression Based on Lipid Metabolism Induced Inflammatory Damage. Arch. Immunol. Ther. Exp..

[34] Nakatani A., Okumura R., Ishibashi A., Okamoto S., Sakaki K., Ito Y., Okuzaki D., Inohara H., Takeda K. (2023). Differential dependence on microbiota of IL-23/IL-22-dependent gene expression between the small- and large-intestinal epithelia. Genes Cells.

[35] Setayesh T., Hu Y., Vaziri F., Wei D., Wan Y.Y. (2024). The spatial impact of a Western diet in enriching Galectin-1-regulated Rho, ECM, and SASP signaling in a novel MASH-HCC mouse model. Biomark. Res..

[36] Vendrov A.E., Lozhkin A., Hayami T., Levin J., Silveira Fernandes Chamon J., Abdel-Latif A., Runge M.S., Madamanchi N.R. (2024). Mitochondrial dysfunction and metabolic reprogramming induce macrophage pro-inflammatory phenotype switch and atherosclerosis progression in aging. Front. Immunol..

