# Supplementary material for: Metabolic Activity in Human Intermuscular Adipose Tissue Directs the Response of Resident PPARγ+ Macrophages to Fatty Acids
Source: Biomedicines. 2024 Dec 25;13(1):10. doi: 10.3390/biomedicines13010010 (PMC11759838; doi:10.3390/biomedicines13010010)
Supplement: Supplementary file 1 [file biomedicines-13-00010-s001.zip › biomedicines-3358720-supplementary.pdf]

Table S1. Participant characteristics.

Participants	Sex	Age (Years)	BMI (kg/m^2)	Type 2 Diabetes (T2D)
P1	Female	22	20.3	No
P2	Female	51	28.9	Yes
P3	Female	55	16.7	No
P4	Female	55	22.6	No
P5	Female	60	22.1	No
P6	Female	64	33.8	No
P7	Male	65	24.2	No
P8	Female	67	46.9	Yes
P9	Male	67	28.6	No
P10	Female	69	22.3	No
P11	Female	70	20	No
P12	Male	72	30.9	No
P13	Female	79	22.8	No
P14	Female	82	30.5	No

Table S2. Correlations between the numbers of PPARγ$^+$ macrophages in IMATs and other macrophage phenotypes in SMFs (upper row) of 14 donors calculated using Spearman's rank analyses.

		CD80$^+$	CD11c$^+$	MARCO$^+$	CD163$^+$	CD206$^+$	PTGER3$^+$
**PPARγ$^+$**	*p*	**0.0359**	0.2926	**0.0303**	0.0721	0.1974	0.2011
	r	-0.5699	-0.3014	-0.5868	-0.4989	-0.3670	-0.3630

The significance levels (*p*) and correlation coefficients (r) are presented. Significant levels of $p \leq 0.05$ are highlighted in bold.

Table S3. Correlations between the number of PPARγ+ macrophages and the expression of 23 chemokines in skeletal muscle tissue samples from12 donors were calculated using Spearman's rank analyses.

		p	*r*
**PPARγ⁺**	Eotaxin (CCL11)	0.7664	0.0979
	GM-CSF	0.4944	0.2193
	GRO alpha (CXCL1)	0.4434	−0.2448
	IFN alpha	0.9425	−0.0246
	IL-1 alpha	0.8346	−0.0699
	IL-13	0.5882	−0.1732
	IL-15	0.8171	0.0769
	IL-17A (CTLA-8)	0.3567	0.2907
	IL-1RA	0.2464	−0.3636
	IL-2	0.9412	0.0254
	IL-21	0.3286	0.3085
	IL-22	0.4640	0.2319
	IL-23	0.4908	0.2187
	IL-27	0.5267	0.2014
	IL-31	0.3391	0.3028
	IL-5	0.1186	0.4769
	IL-7	0.8216	0.0736
	IL-9	0.2627	0.3498
	IP-10 (CXCL10)	0.6673	−0.1399
	MIP-1 alpha (CCL3)	0.1215	−0.4755
	MIP-1 beta (CCL4)	0.4169	−0.2587
	RANTES (CCL5)	0.6192	−0.1608
	TNF beta	0.4975	0.2156

* The significance levels (*p*) and correlation coefficients (r) are presented.

Table S4. Correlations between the mean expression level of VDAC1 in IMATs from 12 donors and the expression of 31 adipokines (upper panel)/chemokines (lower panel) in skeletal muscle tissue samples from12 donors were calculated using Spearman's rank analyses.

		p	*r*
VDAC1	IL-1 beta	0.6231	0.1576
	IL-18	0.8688	−0.0535
	IL-4	0.2310	0.3740
	IL-6	0.2237	0.3795
	IL-8 (CXCL8)	0.7493	−0.1049
	MCP-1 (CCL2)	0.9560	0.0210
	SDF-1 alpha	0.7830	0.0909
	TNF alpha	0.3508	0.2958
	Eotaxin (CCL11)	0.6039	0.1678
	GM-CSF	0.1793	0.4186
	GRO alpha (CXCL1)	0.8692	0.0559
	IFN alpha	0.4453	0.2421
	IL-1 alpha	0.1928	0.4056
	IL-13	0.7769	−0.0919
	IL-15	0.7664	0.0979
	IL-17A (CTLA-8)	0.4663	0.2312
	IL-1RA	0.8861	−0.0490
	IL-2	0.6162	0.1598
	IL-21	0.1346	0.4590
	IL-22	0.1593	0.4338
	IL-23	0.0282	0.6418
	IL-27	0.7676	0.0954
	IL-31	0.0246	0.6549
	IL-5	0.7910	−0.0861
	IL-7	0.0504	0.5752
	IL-9	0.8688	−0.0535
	IP-10 (CXCL10)	0.9913	0.0036
	MIP-1 alpha (CCL3)	0.4573	−0.2378
	MIP-1 beta (CCL4)	0.9388	0.0280
	RANTES (CCL5)	0.9910	−0.0070
	TNF beta	0.1936	0.4028

* The significance levels (*p*) and correlation coefficients (r) are presented. Red labeled values were presented in the diagrams of figure 4a and b.

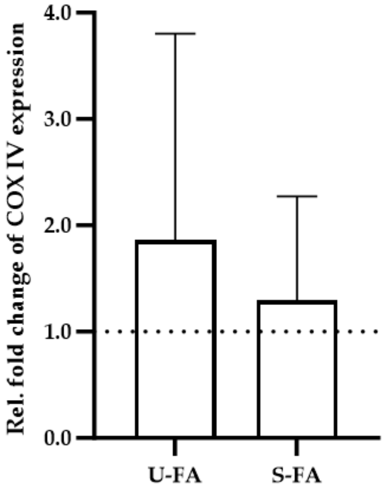

Figure S1. The diagram shows the relative fold change in expression of COXIV (y-axis) in 0.24 mm^2 IMATs of all participants (n = 14) in response to U-FA or S-FAs (x-axis) in vitro. A series of dissected skeletal muscle tissues from all participants were maintained with or without 50 µM concentration of S-FA (C16 or C18) or U-SFA (C16[1] or C18[2]) for 9 or 11 days in vitro. The COXIV expression was analyzed after IF staining with primary antibodies against COXIV followed by staining with fluorescently labeled secondary antibodies and DAPI. The relative fold change in COXIV expression was calculated by normalizing the post-maintenance values to the corresponding premaintenance values. One sample t test and Wilcoxon signed-rank test were employed to assess the level of significance $p \geq 0.29$.

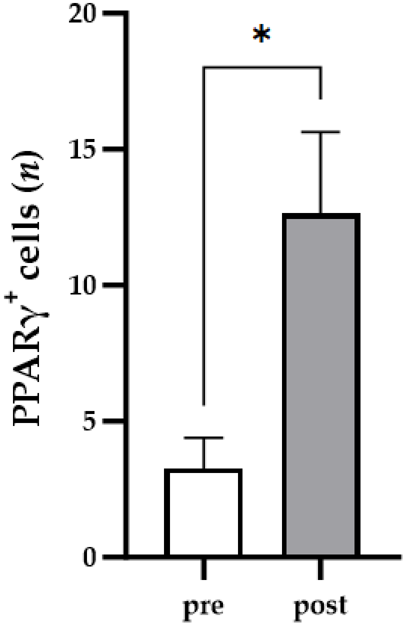

Figure S2. The diagram shows the mean number of PPARγ^{+} macrophages (y-axis) in 0.24 mm^{2} IMATs of participants 1, 10, and 11 (n = 3) before (pre, white bar) and after (post, gray bars) tissue maintenance in vitro. One sample from each participant was preserved in paraffin and sectioned on slides (pre) and additional samples from all donors were maintained in vitro (post). The number of PPARγ+ macrophages were determined after IF staining with primary antibodies against human PPARγ, followed by staining with fluorescently labeled secondary antibodies and DAPI. Two randomly selected microscopic fields of IMAT views (0.24 mm^{2}) were evaluated, rendering mean numbers of PPARγ+ macrophages within the IMATs or SMFs from individual participants. The level of significance (*p*) was analyzed using paired t test.
